# Septic Shock Due to Chyme Recycling in an Extremely Low-Birth-Weight Infant

**DOI:** 10.7759/cureus.91896

**Published:** 2025-09-09

**Authors:** Kazumasa Kitamura, Masashi Zuiki, Tomohiko Imai, Shigeyoshi Aoi, Hiroshi Komatsu

**Affiliations:** 1 Department of Pediatrics, National Hospital Organization Maizuru Medical Center, Maizuru, JPN; 2 Department of Pediatrics, Fukuchiyama City Hospital, Fukuchiyama, JPN; 3 Department of Neonatology, Kyoto Prefectural University of Medicine, Kyoto, JPN; 4 Department of Pediatric Surgery, National Hospital Organization Maizuru Medical Center, Maizuru, JPN; 5 Department of Pediatric Surgery, Tokyo CA Clinic, Tokyo, JPN

**Keywords:** chyme recycling, distal bowel, enterostomy, infant, neonatal, parenteral

## Abstract

Chyme recycling (CR) is an effective therapeutic approach that involves reintroducing bowel contents from the proximal to the distal enterostomy limb and is recognized for its ability to reduce or prevent enterostomy-related complications. Although the benefits of CR are well documented, reports on its potential adverse events remain limited. We report the case of an extremely low-birth-weight infant (ELBWI) who developed septic shock caused by *Klebsiella pneumoniae* immediately after the first CR. The male infant, born at 22 weeks’ gestation via emergency cesarean section, had a birth weight of 496 g. On day 7, he developed focal intestinal perforation and underwent an emergency double-barrel ostomy. CR into the distal bowel was initiated on day 27, after which he developed septic shock. The patient was promptly treated with antibiotic therapy and intensive circulatory support. Blood cultures subsequently confirmed *K. pneumoniae* infection. After a 14-day course of antibiotics, repeat blood cultures were negative. Probiotic infusion into the distal bowel resumed on day 30, and CR was reintroduced on day 70. CR was then continued until ostomy closure on day 123 without further complications. Overall, sepsis may be associated with CR in ELBWIs, necessitating close monitoring after its initiation. Additionally, because of the risk of bacterial proliferation in chyme within the stoma, it is advisable to use freshly collected chyme in the neonatal intensive care unit setting.

## Introduction

Neonates can develop various acute abdominal conditions, including intestinal perforation, meconium ileus, and necrotizing enterocolitis, which may require surgical interventions such as enterostomy. Complications of enterostomy formation include electrolyte and fluid loss, increased dependence on parenteral nutrition (PN), intestinal atrophy, and poor weight gain [1-3].

Chyme recycling (CR), the process of reintroducing bowel contents from the proximal to the distal enterostomy limb, is recognized as a therapeutic approach for effectively reducing or preventing enterostomy-related complications [4-7]. However, standardized, high-quality evidence regarding this intervention remains limited. Reported benefits of CR include improved weight gain, reduced PN dependence, enhanced liver function, normalization of fluid and serum electrolyte levels, promotion of distal gut absorption and maturation, and a reduced size discrepancy between the proximal and distal bowel ends at reversal surgery [3-8]. In contrast, only a few adverse events associated with CR have been reported.

We present the case of an extremely low-birth-weight infant (ELBWI) who developed septic shock due to *Klebsiella pneumoniae *immediately after the first CR.

## Case presentation

The patient was a Japanese male with a birth weight of 496 g. His Apgar scores were 3 at one minute and 5 at five minutes. His mother was a healthy 28-year-old primigravid woman who had undergone cervical cerclage for cervical insufficiency. Preterm premature rupture of membranes occurred at 22 weeks and one day of gestation. Owing to chorioamnionitis and breech presentation, an emergency cesarean section was performed at 22 weeks and three days of gestation.

**Figure 1:**
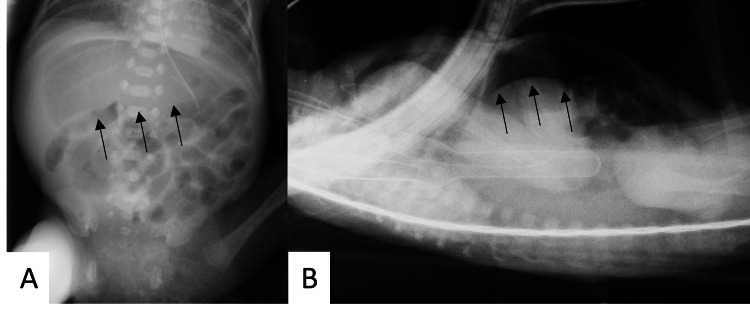
Radiographs demonstrating free intraperitoneal air (A) Supine abdominal radiograph showing free intraperitoneal air. (B) Decubitus abdominal radiograph showing free air layering within the peritoneal cavity.

At birth, the infant was immediately intubated and administered surfactant for respiratory distress syndrome, followed by mechanical ventilation. Enteral feeding with breast milk and probiotics was initiated on day 1 of life. The ductus arteriosus was closed with indomethacin on day 2. On day 3, mild small bowel dilation was observed. Subsequently, free air detected on radiography led to a diagnosis of focal intestinal perforation on day 7 (Figure 1).

**Figure 2:**
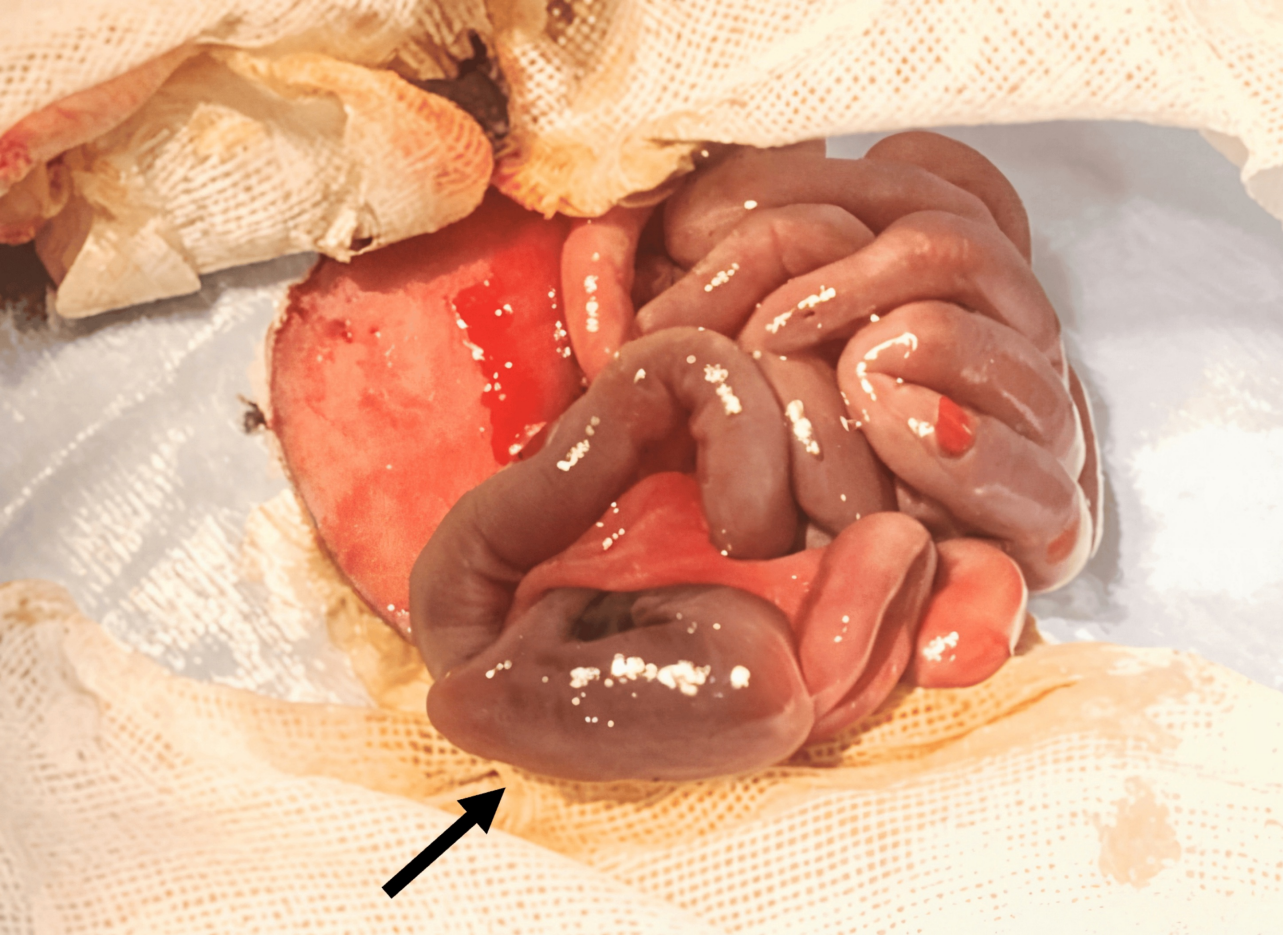
Intraoperative image showing dilation of the intestinal tract proximal to the site of perforation (black arrow)

In this instance, early surgical intervention was considered feasible because of the patient’s clinical stability. Consequently, an emergency laparotomy was performed, which revealed a small intestinal perforation and dilation of the intestinal tract proximal to the perforation site (Figure 2). A double-barrel ostomy was performed immediately.

CR was initiated on day 27 to address inadequate weight gain. Stool samples were collected from the pouch every three hours and reintroduced into the distal bowel. Within hours, the patient developed oliguria and hypotension. Laboratory tests revealed a WBC count of 10,500/µL and an elevated CRP level of 3.32 mg/dL (Table 1).

**Table 1:** Results of laboratory data baso, basophil; CR, chyme recycling; eosin, eosinophil; lymph, lymphocyte; mono, monocyte; Plt, platelet; seg, segmented neutrophils; stab, stab neutrophils

Parameter	Day 26	Day 27 (CR)	Day 28	Day 31	Day 41	Reference range
CRP (mg/dL)	0.01	3.32	16.02	1.87	0.06	0.00-0.14
WBC (/μL)	10,300	10,500	20,400	3,800	4,300	3,500-9,000
Stab (%)	0	6	15	1	0	0-17
Seg (%)	72	73	64	50	56	27-70
Eosin (%)	2	1	0	6	8	0-10
Baso (%)	0	0	0	1	1	0-3
Mono (%)	8	5	17	8	10	0-12
Lymph (%)	17	14	3	30	23	18-59
Plt (×10⁴/μL)	35.3	22	14.3	4.3	26	15.8-34.8
pH	7.352	7.264	7.439	7.303	7.325	7.35-7.45
Lactate (mg/dL)	30	41	11	16	15	9.0-14

No evidence suggested a respiratory or urinary tract infection. Therefore, CR was considered the primary precipitating factor of the sepsis. The patient immediately received antibiotics and intensive circulatory support. The following day, inflammatory markers continued to rise (WBC: 20,400/µL; CRP: 16.02 mg/dL); however, his circulatory status gradually stabilized. A subsequent blood culture identified *K. pneumoniae*. Antibiotic therapy was adjusted to cefotaxime, to which the bacterium was sensitive. Antibiotics were administered for 14 days, resulting in negative blood cultures. On day 30, probiotic infusion into the distal bowel was resumed, and CR was reintroduced on day 70. CR then continued until ostomy closure on day 123, with no further complications and satisfactory weight gain of 2,100 g. Additionally, the luminal diameters of the proximal and distal bowel were comparable (6.0 mm vs. 4.5 mm). The infant was discharged on day 150 without apparent complications.

## Discussion

To the best of our knowledge, this is the first published case describing septic shock associated with CR in an ELBWI. Recently, CR has been increasingly adopted in neonatal intensive care units as a therapeutic approach to reduce or prevent enterostomy-related complications. However, concerns about its safety persist, with reported complications including leakage, effluent reflux, and tube dislodgement [4]. Haddock et al. documented cases of intestinal perforation and recurrent hemorrhage around the mucous fistula site, which required multiple blood transfusions, and one neonatal fatality was reported [7]. Furthermore, Elliott and Walton quantified minor complications, reporting mucous fistula prolapse (3%), enterocutaneous fistula (3%), and redness around the mucous fistula site (13%) [9].

Although no neonatal cases of sepsis have previously been directly linked to CR, its potential risk has been noted [10]. Pataki et al. found that pathogenic bacteria can proliferate in chyme from the proximal stoma, potentially increasing the risk of sepsis if the bag contents are recycled for more than 90 minutes [11]. While universal guidelines are lacking, from a microbiological perspective, we recommend using fresh chyme, as bacteria proliferate easily in stool stored in a warm incubator or at room temperature. In addition, although not performed in this case, it may be advisable to perform a culture test on chyme contents immediately before use. Periodic manual aspiration of the proximal stoma bag and recycling its contents into the distal stoma is a common, simple, and cost-effective method. In recent years, new neonatal CR devices have been developed [12], and safer, more effective approaches are expected to emerge.

The intestinal barrier comprises the outer mucus layer, intestinal epithelial cells, and inner lamina propria [13]. Studies have shown that in premature infants, the mucus layer is reduced, allowing potentially harmful molecules to interact with the underlying intestinal epithelium [14-16]. Increased intestinal bacterial translocation during prematurity triggers a heightened inflammatory response and subsequent intestinal injury [17,18]. Moreover, a recent neonatal study demonstrated that loss of villous structure and increased lymphoid follicles, markers of chronic inflammation and mucosal atrophy, were observed in the distal enterostomy limb without CR [19]. Therefore, meticulous monitoring is essential after CR in ELBWIs, as the distal intestine is particularly prone to histological bacterial translocation.

## Conclusions

Neonates can develop various acute abdominal conditions that may require enterostomy. CR is recognized as a therapeutic approach to effectively reduce or prevent enterostomy-related complications. While CR offers benefits such as improved weight gain and reduced intestinal caliber variation, it also carries a risk of septic shock in infants with extremely low birth weights. Meticulous monitoring throughout the procedure is therefore imperative. Moreover, additional research is required to establish appropriate CR methodologies.
